# Risk Factors for Moderate to Severe Pain during the First 24 Hours after Laparoscopic Bariatric Surgery While Receiving Intravenous Patient-Controlled Analgesia

**DOI:** 10.1155/2019/6593736

**Published:** 2019-10-03

**Authors:** Arissara Iamaroon, Suwimon Tangwiwat, Patchareya Nivatpumin, Thidarat Lertwacha, Piyawadee Rungmongkolsab, Pawinee Pangthipampai

**Affiliations:** Department of Anesthesiology, Faculty of Medicine, Siriraj Hospital, Mahidol University, Bangkok, Thailand

## Abstract

**Objective:**

To investigate the incidence of and risk factors for moderate to severe pain during the first 24 hours after laparoscopic bariatric surgery.

**Materials and Methods:**

This retrospective study included morbidly obese patients who underwent laparoscopic sleeve gastrectomy or Roux-en-Y gastric bypass at a single institution between June 2016 and July 2018. Demographic, clinical, operative, and postoperative pain data from the postanesthesia care unit (PACU) and ward were analyzed. Intravenous patient-controlled analgesia (IV-PCA) was commenced before PACU discharge.

**Results:**

Ninety-seven patients were included. The mean age was 38.60 ± 12.27 years, and the mean BMI was 45.04 ± 8.42 kg/m^2^, and 69% were female. The incidence of moderate to severe pain was 75%. Moderate to severe pain during the first 24 hours was associated with young age, female sex, postoperative administration of NSAIDs, first pain score greater than 3 on arrival at the PACU, and inadequate pain control at PACU discharge. A multivariate analysis revealed that inadequate pain control at PACU discharge was the only factor independently associated with moderate to severe pain during the first 24 hours postoperatively (*p*=0.011). From PACU discharge to the end of postoperative day 3, moderate to severe pain at the end of each 24-hour period was a significant predictor of moderate to severe pain in the subsequent 24-hour period (*p*=0.011, *p* < 0.001, and *p*=0.004, respectively).

**Conclusions:**

Moderate to severe pain was experienced by 75% of patients undergoing laparoscopic bariatric surgery and receiving IV-PCA after PACU discharge. Inadequate pain control at PACU discharge was the only independent risk factor for moderate to severe pain during the first 24 hours postoperatively.

## 1. Introduction

Pain management is challenging in morbidly obese patients [1]. As the first-line drug type for postoperative pain control in these patients is opioids, there is a substantial risk of opioid-induced ventilatory impairment (OIVI) [2]. Moreover, the morbidly obese population has a high prevalence of obstructive sleep apnea (OSA) [3, 4], and the optimal opioid dosages in morbidly obese patients with OSA versus without OSA is not yet known [5]. Concerns about prescribing opioids in morbidly obese patients can lead to inadequate postoperative pain control and resultant adverse consequences.

Intravenous (IV) patient-controlled analgesia (PCA), a pain management strategy that allows patients to titrate their own opioid demand within the analgesic corridor, minimizes the incidence of side effects like OIVI [6, 7]. IV-PCA may be a suitable strategy for postoperative pain control in morbidly obese patients after discharge from the post-anesthesia care unit (PACU). However, to the best of our knowledge, no studies have investigated the efficacy of IV-PCA for controlling pain after laparoscopic bariatric surgery in morbidly obese patients.

The present study aimed to investigate the incidence of and risk factors for moderate to severe pain during the first 24 hours postoperatively in morbidly obese patients who had undergone laparoscopic bariatric surgery and received postoperative IV-PCA before discharge from the PACU.

## 2. Materials and Methods

This retrospective study included morbidly obese patients who underwent either laparoscopic sleeve gastrectomy or Roux-en-Y gastric bypass at a single, large, tertiary referral center between June 2016 and July 2018. Patients were excluded if IV-PCA was not started postoperatively, or if the data relating to their pain scores or the administered analgesics were missing. The study protocol was approved by the Institutional Review Board of our institution.

Records from both the ward staff and the acute pain service were reviewed. The following data were collected: age; sex; BMI; American Society of Anesthesiologists classification; the presence and severity of OSA, diagnosed from the results of a sleep test as mild (Apnea-Hypopnea Index (AHI) ≥5, but <15), moderate (AHI5, but <30), or severe (AHI ≥30); the presence of continuous positive airway pressure use in patients with OSA; and tobacco use (nonsmoking indicated that the patient had stopped using tobacco at least 30 days preoperatively). The surgical data collected were: type of laparoscopic bariatric surgery (Roux-en-Y gastric bypass or sleeve gastrectomy), concomitant surgical procedures, and operative time.

As to information from the PACU, the pain scores at the patients' arrival and discharge and the details of all analgesics given were collected. All patients achieved at least 9 out of a maximum possible score of 10 on our hospital postanesthetic recovery scoring system before discharge from PACU. The scoring elements were movement (0, no movement; 1, purposeless movement; 2, purposeful movement); respiration (0, apnea; 1, dyspnea or limited; 2, deep breath and cough); hemodynamics (0, blood pressure change more than 50% from preoperative baseline; 1, 20%–50% change from baseline; 2, change within 20% baseline); consciousness (0, not responding; 1, arousable on calling; 2, alert and oriented); and color (0, cyanotic; 1, pale, dusky, and jaundiced; 2, normal).

The pain scores and details of all analgesic drugs administered in the period between the PACU discharge and 1 day after discontinuation of the PCA were obtained from ward staff and acute pain service records. Pain severity was rated using a 0–10 numeric rating scale (NRS): 0 indicated no pain, 1–3 mild pain, 4–6 moderate pain, 7–10 severe pain, and 10 maximal pain. Inadequate pain control was defined as moderate to severe pain on the previous day; as the present study focused on the first 24 hours postoperatively, inadequate pain control was defined as moderate to severe pain in the PACU. Nursing ward staff recorded the patient pain score every 4 hours, while the acute pain service recorded the pain score both at rest and during movement once a day or during a follow-up check after adjusting the analgesic regimen. The moderate-to-severe pain group comprised patients that had an NRS >3 at any assessment time during the first 24 hours postoperatively.

All morbidly obese patients who underwent laparoscopic bariatric surgery were prescribed IV-PCA with the setting of bolus morphine 1 mg, a lockout of 5 min, and a 4 h-maximum dose of 30 mg. The daily PCA demand, delivery counts, and cumulative dose were recorded. The dosages of all opioid analgesics were converted to IV morphine equivalents (IV morphine 10 mg is equal to IV fentanyl 100 mcg and equal to IV pethidine 75 mg) [8]. A demand-to-delivery ratio greater than 1.35 was considered a measure of the quality of analgesia, and a more objective method of analgesic assessment than verbal methods of assessment [9]. The severity and management of all complications were analyzed. Sedation was rated as 0 (none), 1 (mild, occasionally drowsy, and easy to arouse), 2 (moderate, constantly or frequently drowsy, and easy to arouse), 3 (severe, somnolent, and difficult to arouse), or S (normal sleep and easy to arouse). Respiratory depression was defined as a respiratory rate lower than 8 breaths/min, and it was rated as absent or present. Nausea, vomiting, and pruritus were rated as 0 (none), 1 (mild, requiring no treatment), 2 (moderate with resolution via medication), or 3 (severe and persistent despite medication). The administration of NSAIDs was recorded; they comprised ketorolac, parecoxib, ibuprofen, and celecoxib. Pre- and intraoperative multimodal anesthesia beyond local anesthetic wound infiltration (which was given in all cases) was recorded, including premedication with analgesic drugs, intraoperative ketamine, and any regional anesthesia.

### 2.1. Sample Size Calculation and Statistical Analysis

The sample size was calculated based on a previously reported incidence of 70% for moderate to severe pain in patients who had undergone laparoscopic bariatric surgery [10]. Using a two-sided test with a 95% confidence interval and an allowable error of 0.1, the calculated sample size was 81 patients. That figure was increased by 20% in 100 patients to compensate for excluded cases.

All statistical analyses were performed using PASW Statistics for Windows, version 18.0 (SPSS Inc., Chicago, IL, USA). Continuous data are presented as the mean ± standard deviation, while categorical data are shown as number and percentage. Data were tested for normal distribution using the Kolmogorov–Smirnov test. As to the univariate analysis, the association of the categorical variables with moderate to severe pain was assessed by the chi-squared test, while the association of the continuous variables with moderate to severe pain was assessed by Student's *t*-test. Variables with a *p* value <0.20 in the univariate analysis were included in the multivariate analysis. A *p* value <0.05 was regarded as statistically significant.

## 3. Results

Ninety-seven patients were included. The mean age was 38.60 ± 12.27 years, and the mean BMI was 45.04 ± 8.42 kg/m2, and 69% were female. The incidences of moderate to severe pain drawn from the records of the acute pain service and the ward staff are presented in Table 1.

There were no significant differences in the demographic and surgical data of the moderate-to-severe pain group versus the no-or-mild pain group (Table 2). There tended to be more females in the moderate-to-severe pain group than in the no-or-mild pain group; however, this difference was not statistically significant. The mean operative time for all patients was 176.65 ± 66.16 minutes.

Data relating to the patients' pain scores and analgesic drugs during the intraoperative, PACU, and postoperative periods are detailed in Table 3. The median pain scores on arrival were 4 (range 0–10) for the no-or-mild pain group vs 7 (0–10) for the moderate-to-severe pain group (*p*=0.038). By comparison, the median pain scores at discharge were 3 (0–6) vs 3 (0–7), respectively (*p*=0.103). The multimodal data for the pre- and intraoperative periods revealed that two patients (20.6%) received a premedication (one received 1 g of paracetamol, while the other received 1 g of paracetamol plus 400 mg of ibuprofen); all patients had wound infiltration with local anesthesia, using 20 ml of 0.5% bupivacaine; one patient received 50 mg of ketamine intraoperatively; and three patients received a bilateral quadratus lumborum block. Only inadequate pain control at PACU discharge was significantly more frequent for the moderate-to-severe pain group than for the no-or-mild pain group. Postoperative NSAID usage tended to be more common in the moderate-to-severe pain group, but the intergroup difference was not significant. The amount of morphine required during the first 24 hours was 20.60 ± 15.77 vs 29.17 ± 18.86 mg (*p*=0.44) for the no-or-mild pain and the moderate-to-severe pain groups, respectively.

The univariate analysis revealed that the two groups significantly differed regarding age, female sex, postoperative NSAID usage, patients with NRS >3 on arrival at the PACU, and inadequate pain control at PACU discharge (*p* < 0.2; Tables 2 and 3). When those factors were included in the multivariate analysis, only inadequate pain control at PACU discharge was identified as an independent risk factor for moderate to severe pain during the first 24 hours postoperatively (Table 4).

**Figure 1:**
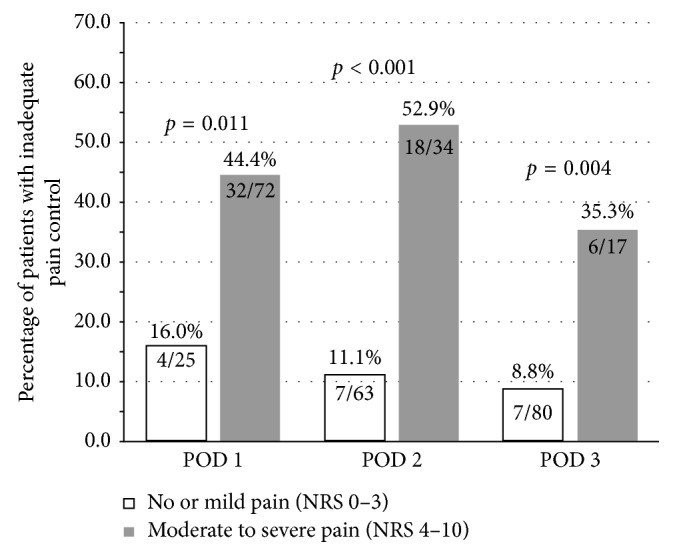
Percentage of patients with inadequate pain control at the end of the previous 24-hour period on postoperative days 1–3. *p* < 0.05 indicates statistical significance. Abbreviations: POD, postoperative day; NRS, numeric rating scale.

As to the effect of inadequate pain control at the end of the previous day on the pain status of the following day, there were significant differences between the two groups at PACU discharge (*p*=0.011), at the end of the first 24 hours postoperatively (*p* < 0.001), and at 48 hours postoperatively (*p*=0.004; Figure 1).

The adverse effects and complications resulting from IV-PCA opioids did not differ between the two groups (Table 5). No patient experienced respiratory depression. The lengths of hospital stay were also similar for the groups.

## 4. Discussion

The present study revealed a high incidence (74.2%) of moderate to severe pain in morbidly obese patients after laparoscopic bariatric surgery. In 1997, it was proposed that less than 5% of patients should experience pain after surgery by 2002^11^; this proclamation was intended to encourage a worldwide increase in the standard of patient care [11]. We used the pain scores from ward staff records rather than from the acute pain service records to divide the study patients into the two groups for analysis, given that the ward nurses perform pain assessments every 4 hours rather than once daily. However, the pain scores from the ward staff records were similar to the pain scores during movement from the acute pain service data. We believe that the pain score during movement during the postoperative period is important because adequate pain control during patient movement results in earlier ambulation and better postoperative outcomes [12].

Several studies have reported that younger age and female sex increase the risk of postoperative pain [13–16]; however, other studies have reported contrary findings [17, 18]. In the present study, the multivariate analysis revealed no significant differences in age or sex between those with moderate to severe pain versus patients with no to mild pain postoperatively. More patients received NSAIDs in the moderate-to-severe pain group than in the no-or-mild pain group, which seems to contradict the fact that NSAIDs are used in multimodal analgesic protocols. However, as there was a concern that NSAID administration may cause postoperative gastric leakage and marginal ulcers, NSAIDs were only prescribed for patients with uncontrollable pain. Although NSAID usage was significantly associated with moderate to severe pain in the univariate analysis, this factor did not remain significant in the multivariate analysis.

Multimodal analgesia combines more than one type of analgesic to achieve pain control, while simultaneously reducing opioid consumption and opioid-related side effects. Multimodal analgesia regimens are the core of Enhanced Recovery after Surgery protocols [19]. In the current study, only 38.1% of the patients received a multimodal analgesia; however, multimodal strategies should be used throughout the perioperative period, i.e., pre-, intra-, and postoperatively. Although the benefits of multimodal treatment are well recognized, the use of multimodal anesthesia has been limited [20]. Multimodal analgesic protocols should be strictly implemented as a mandatory hospital policy to reduce opioid use, reduce opioid-related side effects, and improve pain relief in morbidly obese patients [21].

The multivariate analysis revealed that the only independent risk factor for pain after laparoscopic gastric bypass surgery was inadequate pain control upon PACU discharge. In our study, inadequate pain control was defined as an NRS >3 during the previous day [22]. IV-PCA is now more commonly used in demand mode only with no background infusion [18], since the routine addition of a continuous infusion has no beneficial effects for average opioid-naive patients. More specifically, the addition of a background infusion does not always reduce the number of demands made by a patient, nor does it result in better analgesia and improved sleep patterns. Moreover, it may increase the total amount of opioid delivered, and it significantly increases the risk of OIVI [23]. The principle of using PCA in demand mode only is that patient comfort must be accomplished before the commencement of PCA. A loading dose of opioid is given until a reported pain score of 3 out of 10 is achieved before PCA is started [24] because this helps achieve the minimum effective analgesic opioid concentration. This principle helps to explain why inadequate pain control at PACU discharge was a strong predictor of moderate to severe pain within the 24 hours after PACU discharge [1]. This is despite obese patients being more sensitive to opioids and requiring much lower opioid doses to achieve similar analgesic endpoints. This awareness may lead to suboptimal treatment by virtue of inter individual variations in opioid requirements. Therefore, an individual titration would be the best way to manage pain, especially with challenging obese patients. This narrow, therapeutically effective opioid dose could be administered safely in the PACU with close monitoring and an adequate number of caregivers. The present results also revealed that inadequate pain control in the previous period (PACU discharge, 24 hours postoperatively, or 48 hours postoperatively) resulted in moderate to severe pain during the subsequent period.

IV-PCA is not suitable for all patients. Optimal pain control requires the tailoring of treatment to the individual. The optimal bolus dose is that which results in good pain relief with minimal adverse effects. Therefore, the initial dose may need to be adjusted so that IV-PCA meets the requirements of each individual patient. IV-PCA is not a “one size fits all” or “set and forget” therapy [25]. We also evaluated the demand-to-delivery ratios of the moderate-to-severe pain group and the no-or-mild pain group as this ratio is often used as an indicator of pain and the quality of analgesia [9]. The demand-to-delivery ratio points out a high number of unsuccessful demands which may reflect the need for more opioid. However, our results revealed no significant difference in the groups' ratios and more than half of the patients in each group had a demand-to-delivery ratio >1.35. This may be explained by patient anxiety, patient confusion, and/or inappropriate patient use of IV-PCA.

Although we found no significant differences in the postoperative complications of the groups, inadequate postoperative pain control has previously been associated with immune deficiency, slower wound healing, and the development of chronic pain [10]. Moreover, there have been reports of psychological effects, such as a reduced quality of life, sleep-related problems, and feelings of demoralization and anxiety [26, 27]. Unrelieved pain has also been found to be significantly associated with higher economic costs [27].

## 5. Limitations

This study has some limitations. Firstly, this was a retrospective study that analyzed data from a single center. Furthermore, we had no information as to whether patients were trained in how to correctly use IV-PCA. Future research should attempt to identify barriers to the effective management of acute pain, and the consequences of inadequate pain management in morbidly obese patients.

## 6. Conclusions

The incidence of moderate to severe pain in patients who underwent laparoscopic bariatric surgery and received IV-PCA after PACU discharge was 75%. Inadequate pain control at discharge from the PACU was the only independent risk factor for moderate to severe pain during the first 24 hours postoperatively. Caregivers should improve pain management in this patient population to improve the level of patient care.

## Figures and Tables

**Table 1:** Pain scores during the first 24 hours after laparoscopic bariatric surgery (*n* = 97).

Pain data	No or mild pain (NRS 0–3)	Moderate to severe pain (NRS 4–10)
Recorded by the acute pain service		
Pain at rest	65 (67.0%)	32 (33.0%)
Pain during movement	23 (23.7%)	74 (76.3%)
Recorded by the ward nursing staff	25 (25.8%)	72 (74.2%)

Values are given as *n* (%). Abbreviation: NRS, numeric rating scale.

**Table 2:** Demographic and surgical data related to pain severity.

Data	No or mild pain (NRS 0–3; *n* = 25)	Moderate to severe pain (NRS 4–10; *n* = 72)	*p* value
Age (years)	41.60 ± 12.34	37.56 ± 12.16	0.157
Female sex	14 (56.0%)	53 (73.6%)	0.101
BMI (kg/m^2^)	43.56 ± 6.14	45.55 ± 9.06	0.311
ASA classification			0.613
2	5 (20.0%)	18 (25.0%)	
3	20 (80.0%)	54 (75.0%)	
Obstructive sleep apnea			0.796
None	12 (48.0%)	37 (51.4%)	
Mild	2 (8.0%)	6 (8.3%)	
Moderate	2 (8.0%)	9 (12.5%)	
Severe	9 (9.0%)	20 (27.8%)	
CPAP (yes)	12 (48.0%)	31 (43.1%)	0.668
Type of operation			0.514
Roux-en-Y	12 (48.0%)	40 (55.6%)	
Sleeve gastrectomy	13 (52.0%)	32 (44.4%)	
Concomitant operations			0.586
Hiatal hernia	1 (4.0%)	1 (1.4%)	
LC	3 (12.0%)	5 (6.9%)	
LC and hiatal hernia	0 (0.0%)	2 (2.8%)	
Operative time (min)	171.92 ± 67.03	178.29 ± 66.25	0.680

*p* < 0.05 indicates statistical significance. Values are given as *n* (%) or mean ± standard deviation. Abbreviations: NRS, numeric rating scale; BMI, body mass index; ASA, American Society of Anesthesiologists; CPAP, continuous positive airway pressure; LC, laparoscopic cholecystectomy.

**Table 3:** Pain and analgesic data during intraoperative, PACU, and postoperative periods.

Data	No or mild pain (NRS 0–3; *n* = 25)	Moderate to severe pain (NRS 4–10; *n* = 72)	*p* value
*Intraoperative*			
Intraoperative morphine (mg)	19.56 ± 5.39	20.62 ± 6.43	0.463
Intraoperative NSAIDs	4 (16%)	9 (12.5%)	0.735
Multimodal analgesia	7 (28.0%)	27 (37.5%)	0.391
*PACU*			
NRS >3 on arrival	14 (56%)	55 (76.4%)	0.053
PACU morphine (mg)	1.18 ± 1.80	1.69 ± 2.22	0.305
PACU NSAIDs	1 (4%)	8 (11.1%)	0.439
Inadequate pain control at PACU discharge	4 (16.0%)	32 (44.4%)	**0.011**
*Postoperative*			
Demand-to-delivery ratio	2.08 ± 1.79	1.89 ± 0.97	0.523
Demand-to-delivery ratio >1.35	14 (56.0%)	45 (62.5%)	0.566
Postoperative NSAIDs	4 (16.0%)	24 (33.3%)	0.099

*p* < 0.05 indicates statistical significance. Values are given as *n* (%) or mean ± standard deviation. Abbreviations: PACU, postanesthesia care unit; NRS, numeric rating scale; NSAIDs, nonsteroidal anti-inflammatory drugs.

**Table 4:** Multivariate analysis of factors associated with moderate to severe pain during the first 24 hours postoperatively.

Factors	Adjusted OR (95% CI)	*p* value
Age	0.96 (0.92–1.01)	0.104
Female sex	2.68 (0.91–7.91)	0.075
Postoperative NSAIDs	3.29 (0.91–11.85)	0.069
Patients with NRS >3 on arrival at PACU	1.03 (0.31–3.46)	0.961
Inadequate pain control at PACU discharge	4.90 (1.44–16.69)	**0.011**

*p* < 0.05 indicates statistical significance. Abbreviations: OR, odds ratio; CI, confidence interval; NSAIDs, nonsteroidal anti-inflammatory drugs.

**Table 5:** Sedation scores, adverse effects, and lengths of hospital stay of the two groups.

Data	No or mild pain (NRS 0–3; *n* = 25)	Moderate to severe pain (NRS 4–10; *n* = 72)	*p* value
Sedation score			0.601
0	23 (92.0%)	69 (95.8%)	
1	2 (8.0%)	3 (4.2%)	
2	0 (0.0%)	0 (0.0%)	
Respiratory depression	0 (0.0%)	0 (0.0%)	
PONV			0.654
0	18 (72.0%)	56 (77.8%)	
1	7 (28.0%)	15 (20.8%)	
2	0 (0.0%)	1 (1.4%)	
Pruritus			0.320
0	24 (96%)	64 (88.9%)	
1	0 (0.0%)	6 (8.3%)	
2	1 (4.0%)	2 (2.8%)	
LOS	5.08 ± 1.15	5.14 ± 1.04	0.813

*p* < 0.05 indicates statistical significance. Values are given as *n* (%) or mean ± standard deviation. Abbreviations: PONV, postoperative nausea and vomiting; LOS, length of hospital stay.

## Data Availability

The data from this clinical study will be available in SPSS format for sharing provided all conditions stipulated by the local university are met.
